# Extending Association Rule Mining to Microbiome Pattern Analysis: Tools and Guidelines to Support Real Applications

**DOI:** 10.3389/fbinf.2021.794547

**Published:** 2022-01-10

**Authors:** Agostinetto Giulia, Sandionigi Anna, Bruno Antonia, Pescini Dario, Casiraghi Maurizio

**Affiliations:** ^1^ Department of Biotechnology and Biosciences, University of Milano-Bicocca, Milan, Italy; ^2^ Quantia Consulting Srl, Milan, Italy; ^3^ Department of Statistics and Quantitative Methods, University of Milano-Bicocca, Milan, Italy

**Keywords:** pattern mining, microbiome data, DNA metabarcoding, microbiome patterns, machine learning, association rule mining

## Abstract

Boosted by the exponential growth of microbiome-based studies, analyzing microbiome patterns is now a hot-topic, finding different fields of application. In particular, the use of machine learning techniques is increasing in microbiome studies, providing deep insights into microbial community composition. In this context, in order to investigate microbial patterns from 16S rRNA metabarcoding data, we explored the effectiveness of Association Rule Mining (ARM) technique, a supervised-machine learning procedure, to extract patterns (in this work, intended as groups of species or taxa) from microbiome data. ARM can generate huge amounts of data, making spurious information removal and visualizing results challenging. Our work sheds light on the strengths and weaknesses of pattern mining strategy into the study of microbial patterns, in particular from 16S rRNA microbiome datasets, applying ARM on real case studies and providing guidelines for future usage. Our results highlighted issues related to the type of input and the use of metadata in microbial pattern extraction, identifying the key steps that must be considered to apply ARM consciously on 16S rRNA microbiome data. To promote the use of ARM and the visualization of microbiome patterns, specifically, we developed microFIM (microbial Frequent Itemset Mining), a versatile Python tool that facilitates the use of ARM integrating common microbiome outputs, such as taxa tables. microFIM implements interest measures to remove spurious information and merges the results of ARM analysis with the common microbiome outputs, providing similar microbiome strategies that help scientists to integrate ARM in microbiome applications. With this work, we aimed at creating a bridge between microbial ecology researchers and ARM technique, making researchers aware about the strength and weaknesses of association rule mining approach.

## 1 Introduction

Studying microbiome patterns is now a hot-topic in different fields of application (Kyrpides et al., 2016; Wood-Charlson et al., 2020). From ecology to medicine, microbiomes are undoubtedly a cornerstone of research, acknowledged as being key participants in all ecosystems, including the human one (Duvallet et al., 2017; Layeghifard et al., 2017). In recent years, DNA sequencing strategies have become one of the main sources for studying microbial communities (Wood-Charlson et al., 2020). Further, 16S rRNA metabarcoding is currently the preferential method to obtain great amounts of information in a time and cost effective manner (Wood-Charlson et al., 2020), becoming one of the primary sources of data regarding microbiome studies (Gonzalez et al., 2018; Knight et al., 2018; Bokulich et al., 2020; Mitchell et al., 2020).

In this context, data mining approaches seem to be newfangled solutions for disclosuring and understanding microbial ecosystems (Wood-Charlson et al., 2020; Galimberti et al., 2021; Ghannam and Techtmann, 2021). Spanning from classification and signature extraction to interaction and trait associations (Pasolli et al., 2016; Qu et al., 2019), data mining strategies can identify hidden patterns that may help to predict biological functions (Noor et al., 2019; Thomposon et al., 2019). Investigating patterns and exploring their role in functional and predictive aspects are now pivotal to proxy the knowledge of microbial associations, both disentangling interactions and niche specialization (Chaffron et al., 2010; Faust and Raes, 2012; Ma et al., 2020).

Considering the size and complexity of High-Throughput Sequencing (HTS) 16S rRNA metabarcoding data, interpretation and summarization are not straightforward (Naulaerts et al., 2015) and, for this reason, pattern mining strategies have become essential for researchers to disentangle the high amount of information (Kyrpides et al., 2016; Wood-Charlson et al., 2020; Ghannam and Techtmann, 2021).

Recently, association rule mining (ARM) emerged as a promising technique to study microbiome patterns (Naulaerts et al., 2015; Tandon et al., 2016). Specifically, Tandon et al. (2016) have demonstrated the potentials of this technique on two microbiome datasets, in particular the HMP dataset (Turnbaugh et al., 2007) and two prebiotic studies (Kato et al., 2014; Xiao et al., 2014). From the classic application on market basket problems (Agrawal et al., 1993), association rule mining started to be applied to answer a wide range of biological questions. From annotation tasks (Manda et al., 2012; Manda et al., 2013; Manda, 2020) to protein interaction networks (Koyuturk et al., 2006), ARM was applied to a wide range of research fields, including genetics (Carmona-Saez et al., 2006; Alves et al., 2010; Karpinets et al., 2012; Ong et al., 2020), molecular biology (Agapito et al., 2015; Boutorh and Guessoum, 2016; Naulaerts et al., 2016), and biochemical disciplines (Yoon and Lee, 2011; Zhou et al., 2013; Naulaerts et al., 2016). Noticeably, the expression ‘association rule mining’ comprehends two main phases: 1) frequent itemset mining, the extraction of patterns intended as elements often co-occur together in a dataset (Agrawal et al., 1993), and 2) rule calculation, to identify strong association between patterns previously extracted (Agrawal et al., 1993).

Despite the apparent simplicity of use, large datasets can produce high numbers of patterns, making their extraction difficult (Agrawal et al., 1993; Han et al., 2004; Karpinets et al., 2012; Naulaerts et al., 2015). Beside several algorithms have been developed to better capture reliable patterns, as for example Eclat (Agrawal et al., 1996), FP-Growth (Han et al., 2004) or Apriori (Agrawal et al., 1993), avoiding uninformative or spurious information is still a current issue (Naulaerts et al., 2015). Interesting measures such as support (frequency of a pattern) or pattern length are pivotal to control the generation and the evaluation of patterns discovered (Agrawal et al., 1993; Karpinets et al., 2012; Naulaerts et al., 2015). Still, a few issues exist in setting these parameters (Naulaerts et al., 2015). Considering the support, setting a low value leads to a high amount of patterns, difficult to explore and visualize. At the same time, setting a high support value can be detrimental for finding rare but informative patterns. Over and above, researchers try to identify metrics that can be used to pinpoint patterns of interest (and so called “interest measures”). In detail, several metrics have been implemented (Tan et al., 2002; Omiecinski, 2003; Franceschini et al., 2012; Tang et al., 2012), as for example lift or maximal entropy (Tatti and Mampaey, 2010; Hussein et al., 2015). Nevertheless, extracting effective information is not an easy task as the definition of interestingness is strictly associated with the biological question and the research field under study (Koyutürk et al., 2006; Karpinets et al., 2012; Naulaerts et al., 2015). Considering the rule calculation phase, issues regarding the evaluation of reliable rules remain (Karpinets et al., 2012; Naulaerts et al., 2015). In general, taking into account previous works, the most widely used parameters to evaluate both patterns and rules are support and confidence, where confidence is a measure that describes the strength of the association between the two elements of the rule (Naulaerts et al., 2015).

Recently, different works related to pattern mining applied to microbiome studies were published, such as MITRE (Bogart et al., 2019), MANIEA framework (Liu et al., 2021) and the work of Tandon et al. (2016). Nevertheless, as also highlighted by the work of Faust (2021), applying such an algorithm still has its limitations and, despite the efforts of recent works, guidelines for microbiome data applications have not been completely defined (Naulaerts et al., 2015; Faust, 2021). Different libraries have been implemented, such as pyfim (Muino and Borgelt, 2014), mlxtend (Raschka, 2018) and arules (Hahsler et al., 2011). A few frameworks have been recently developed and applied on real case studies (Tandon et al., 2016; Liu et al., 2021). However, tests to establish specific best practices for 16S rRNA metabarcoding data do not exist.

Apart from the availability of tools, the application of pattern mining to study microbiome patterns must consider the intrinsic biological aspect of microbiome data (Balint et al., 2016; Gloor et al., 2017). Beside the issues related to species abundances that should be filtered to obtain a solid input dataset, also metadata composition and taxonomy level should be considered. Further, microbiome matrices can be large and complex: composed of thousands of taxa and hundreds of samples (Faust, 2021; Ghannam and Techtmann, 2021), microbiome data can affect pattern mining approaches, sometimes obliging to set high but improper interest measures. This last point is crucial if we consider that 16S rRNA metabarcoding data can describe putative ecological properties and sparse microbial associations (Faust, 2021).

Given these premises, our work wants to shed light on the strengths and weaknesses of pattern mining strategy into the study of microbial patterns, in particular from 16S rRNA microbiome datasets. In detail, we show pitfalls of ARM applied on real case studies, highlighting issues related to the type of input and the use of metadata. Then, we identify the key steps that must be considered to apply ARM consciously on 16S rRNA microbiome data. Moreover, to facilitate the integration of ARM technique into microbiome pipeline, we developed microFIM (microbial Frequent Itemset Mining), a versatile user-friendly and open source Python tool that promotes the use of ARM integrating common microbiome practices, such as taxa tables and distance matrix visualizations. Besides the conventional parameters, microFIM implements interest measures to remove spurious information. Moreover, it merges the results of ARM analysis with the typical microbiome outputs, aiming at creating a bridge between microbial ecology research and ARM technique.

## 2 Materials and Methods

**FIGURE 1:**
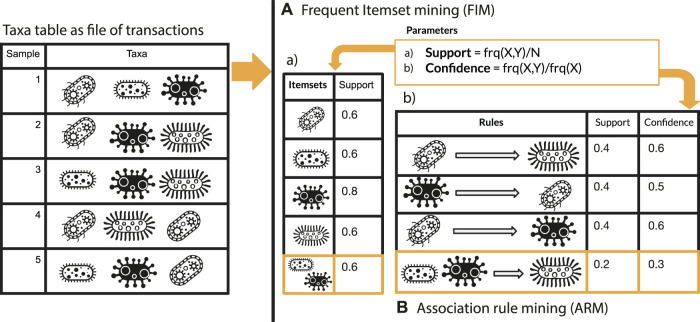
Graphical overview of Frequent Itemset Mining **(A)** and Association Rule mining **(B)** approach integrated with elements related to microbiome analysis.

This section comprehends two main paragraphs: 1) description of microFIM (microbial Frequent Itemset Mining) tool to promote microbiome pattern exploration with two simulated dataset and 2) microFIM analysis on real case microbiome datasets to highlight ARM potentials and caveats. microFIM was developed on the basis of Frequent Itemset Mining (Naulaerts et al., 2015), in which patterns of elements that co-occur can be extracted from a transactional dataset, typically (Naulaerts et al., 2015). A pattern (or itemset) is called frequent if its support value within the dataset is greater than a given minimal support threshold. For an overview of the method and its translation in terms of bacterial composition instead of elements, please see Figure 1. A complete description of the approach with formalized expression can be found in the works of Tan et al., 2002 (Chapter 6), Goethals, 2005, and Naulaerts et al. (2015).

### 2.1 microFIM Implementation

To promote and integrate the use of ARM in microbiome studies, we developed microFIM (microbial Frequent Itemset Mining), a versatile open-source user-friendly tool implemented in Python (v. > 3; https://github.com/qLSLab/microFIM).

microFIM receives as input the taxa table and the metadata file used during the microbiome bioinformatic analysis. In particular, a taxa table is composed of rows and columns representing the taxa and their abundances for each sample. It derives from the conversion of the BIOM file into a CSV or TSV file (https://biom-format.org/). In general, considering the well-established QIIME2 microbiome platform (https://qiime2.org/; Bolyen et al., 2018), complete frameworks and scripts to analyse and obtain taxa tables are implemented.

To promote the usage to a wider group of researchers, the tool can be used both *via* Python functions and running the pre-settled scripts, which allow interactivity through the command-line, avoiding coding implementations. To favor easy integration in Python scripting and future implementation of additional functions and metrics, Python functions were divided into thematic sections. microFIM is composed by six main steps: 1) filtering taxa table with metadata, 2) converting taxa table into a transactional database to be read by ARM algorithms, 3) extract microbiome patterns, 4) calculate additional interest measures to evaluate the patterns extracted, 5) create the pattern table (a taxa table improved with patterns, presence-absence information among samples and interest measures) and 6) visualization of results.

Template files are provided to run microFIM scripts. Considering interest measures, we integrated support, pattern length and all-confidence metrics, which generates “hyperclique patterns” (Agrawal et al., 1993; Tan et al., 2002; Omiecinski, 2003; Xiong et al., 2006). Considering a pattern “X” composed of different items, all-confidence is calculated as the ratio between the support of “X” and the highest support retrieved from the elements of the pattern “X.” For example, a pattern X is composed of three elements that, considering the entire dataset, have the following support threshold: 0.3, 0.6 and 0.8. Overall, the pattern X has a support of 0.3. All-confidence will be calculated as the ratio between the support of X—0.3—and the higher support within X—0.8, resulting in 0.37. All-confidence, in this way, is defined as the smallest confidence of all rules which can be produced from a pattern, i.e., all rules produced from a pattern will have a confidence greater or equal to its all-confidence value (Tan et al., 2002; Omiecinski, 2003). In detail, confidence is an indication of how often a rule has been found to be true, so it is considered as a measure of rule reliability (Hornik et al., 2005; Hahsler et al., 2011; Naulaerts et al., 2015).

In order to show the usage and the potentials of microFIM, we tested the tool on simulated matrices (available in Supplementary Tables S1, S2) and on real case studies. In particular, the cases selected are: 1) the ECAM dataset (Bokulich et al., 2016), 2) the vaginal microbiome dataset of Ravel et al. (2011) and 3) the Montassier dataset (Montassier et al., 2016). Details about the application of microFIM on real case studies are described in the next sections. Parameters used to run microFIM on simulated matrices are the following: 0.3 as minimum support threshold, a minimum of two elements and a maximum of 10 to extract patterns.

In the Results section, a complete scheme of the tool is provided. microFIM is mainly based on four Python libraries: fim (Muino and Borgelt, 2014), Pandas (McKinney, 2010; Reback et al., 2020), Numpy (Harris et al., 2020), and plotly (https://plotly.com/). It is available as a conda environment (https://docs.anaconda.com/; Anaconda Software Distribution, 2020) and all the details about tutorials and installation are available in our Github repository (https://github.com/qLSLab/microFIM). Python notebooks and an example of microFIM usage *via* scripting are also reported in the repository. In general, beside the focus of this work, microFIM may potentially be used for a wide range of applications. As the primary resource input consists in a matrix describing the presence-absence of an element (rows) in a dataset (columns, representing samples), fields of study in which it can be applied may be various, also merely consider the analysis of OTU (Operational Taxonomic Unit) or ESV (Exact Sequence Variants) instead of taxa (Schloss and Westcott, 2011; Callahan et al., 2017) of 16S rRNA metabarcoding data.

### 2.2 Real Case Studies Analysis

To show the caveats and potentials of association rule mining, we used microFIM on three real case studies: the ECAM dataset (Early Childhood Antibiotics and the Microbiome; Bokulich et al., 2016), the vaginal microbiome case study of Ravel et al. (2011) and Montassier case study (Montassier et al., 2016). Different input types were selected based on taxonomy level and metadata composition. In detail, the ECAM dataset collects a total of 875 samples, describing the gut microbiome of the first 2 years of life of 43 infants. Presence-absence tables were created taking account of the taxonomic rank. In particular, we used: 1) the taxa table obtained directly from QIIME2 datasets (Bolyen et al., 2018) in which only taxa assigned to genus level, with a relative abundance > 0.1% in more than 15% of samples, are considered (Input 1—data are available in Supplementary Table S3); 2) family table obtained from collapsing the previous Input 1 *via* QIIME2 plugins (https://github.com/qiime2/q2-taxa; Input 2—Supplementary Table S4); 3) a taxa table consisting only of taxa with complete taxonomy at the genus level (Input 3—Supplementary Table S5). Metadata as type of delivery and antibiotic exposition were considered to evaluate patterns extraction.

Considering the vaginal microbiome dataset (Ravel et al., 2011), we obtained from MLRepo repository (Vangay et al., 2019) the taxa table obtained via the MLRepo pipeline (Vangay et al., 2019). The dataset collects 388 samples, investigating the vaginal microbiome of 396 asymptomatic North American women. Additional presence-absence tables were created taking account of the taxonomic rank, in particular from the original dataset obtained from MLRepo, also family and genus levels were considered. Low and high nugent score values (a scoring system for vaginal swabs to diagnose bacterial vaginosis) were considered for the evaluation regarding metadata filtering.

Finally, the dataset of Montassier et al. (2016) was included. The dataset collects 28 samples from patients with non-Hodgkin lymphoma undergoing allogeneic hematopoietic stem cell transplantation (HSCT) in order to identify microbes that predict the risk of BSI (bloodstream infection). OTU table and taxa table obtained with MLRepo pipeline were selected (Vangay et al., 2019).

For the ECAM and Ravel et al. (2011) datasets, minimum support threshold of 0.2, minimum length of 3 and a maximum length of 15 elements were used. Montassier et al. (2016) datasets were analysed considering a minimum support of 0.9, a minimum length of 5 and a maximum length of 10. After pattern extraction, interest measures as support, pattern length and all-confidence were calculated (Tan et al., 2002; Omiecinski, 2003; Xiong et al., 2006). Distributions of number of patterns, length and support were evaluated considering both ARM analysis and interest measures filtering. A minimum of 0.5 and 0.8 of all-confidence were used to evaluate hypercliques patterns (Tan et al., 2002; Omiecinski, 2003; Xiong et al., 2006). Considering metadata filtering, pattern extraction was performed with the previous settings. A minimum of 0.8 of all-confidence was used to evaluate hypercliques patterns (Tan et al., 2002; Omiecinski, 2003; Xiong et al., 2006). Visualizations were created with plotly and pandas Python libraries. Both datasets, results and metadata files are available in Supplementary Material.

## 3 Results

### 3.1 microFIM Tool: Extending Association Rule Mining to Microbiome Pattern Analysis

Association rule mining demonstrates its useful properties in different contexts (Naulaerts et al., 2015; Tandon et al., 2016). To promote the use of ARM in the microbial community field, we implemented microFIM, a versatile open-source project developed in Python and freely available at https://github.com/qLSLab/microFIM.

**FIGURE 2:**
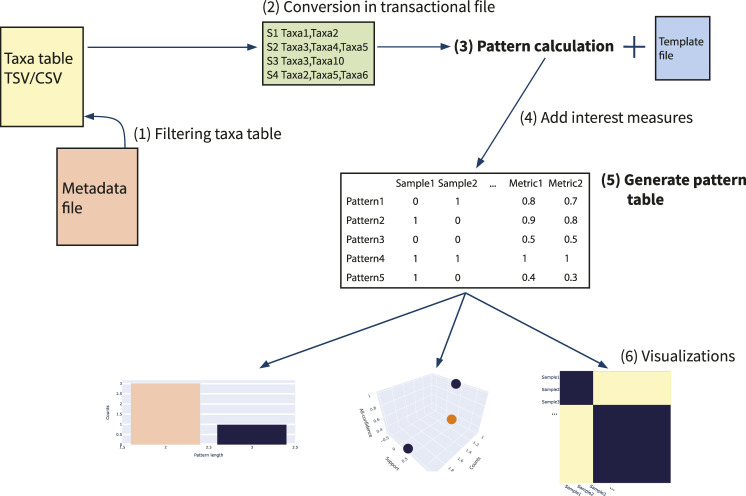
Scheme of microFIM framework. 1) Filtering taxa table; 2) Conversion of taxa table into transactional file; 3) Extract patterns with template file filled with minimum support threshold, minimum and maximum length; 4) Adding of interest measures as support, pattern length and all-confidence (Omiecinski, 2003; Xiong et al., 2006); 5) Generating pattern table, composed by presence-absence of patterns within samples and interest measures; 6) Generating visualizations.

In this section, we explain the framework of usage, the main steps of pattern extraction and filtering and insights of visualizations available. In addition, two main examples are reported, in order to show the workflow of the tool. In Figure 2 a scheme of microFIM framework is reported. In particular, microbiome data (taxa table) can be filtered (step 1) and then converted into a transactional dataset (step 2), in order to be read as input by association rule mining algorithm. Subsequently, patterns can be generated setting parameters via a template file to be filled (tutorials and templates are available at https://github.com/qLSLab/microFIM) (step 3). In detail, minimum support threshold, minimum and maximum length of patterns must be specified. Pattern extraction was implemented via pyfim library (Muino and Borgelt, 2014). At this stage, the default algorithm used is Eclat (Muino and Borgelt, 2014), but other algorithms are available within the pyfim library (Apriori or FP-Growth; Muino and Borgelt, 2014). The set of interest measures initially calculated are “support” and “pattern length” (which describes the number of elements belonging to a pattern). Further, other interest measures are added (step 4) and can be used to filter patterns. In microFIM implementation, all-confidence interest measure was included, in order to help remove spurious information (Tan et al., 2002; Omiecinski, 2003; Xiong et al., 2006). As described in Section 2, all-confidence can be used to set the smallest confidence of all rules that can be produced from a pattern, i.e., all rules produced from the pattern will have a confidence greater or equal to its all-confidence value, creating the basis for rule reliability exploration at the pattern level (Tan et al., 2002; Hornik et al., 2005; Omiecinski, 2003; Xiong et al., 2006; Hahsler et al., 2011; Naulaerts et al., 2015).

The main result of this step is the creation of the pattern table (step 5). Conceptually similar to the microbiome taxa table, the pattern table described the presence of a pattern for each sample, integrating the interest measures previously calculated (step 4). microFIM visualizations comprehend distributions of patterns considering support, length and interest measure values. To describe the relationships between samples considering patterns found, a Jaccard matrix can be also obtained and visualized (step 6).

**FIGURE 3:**
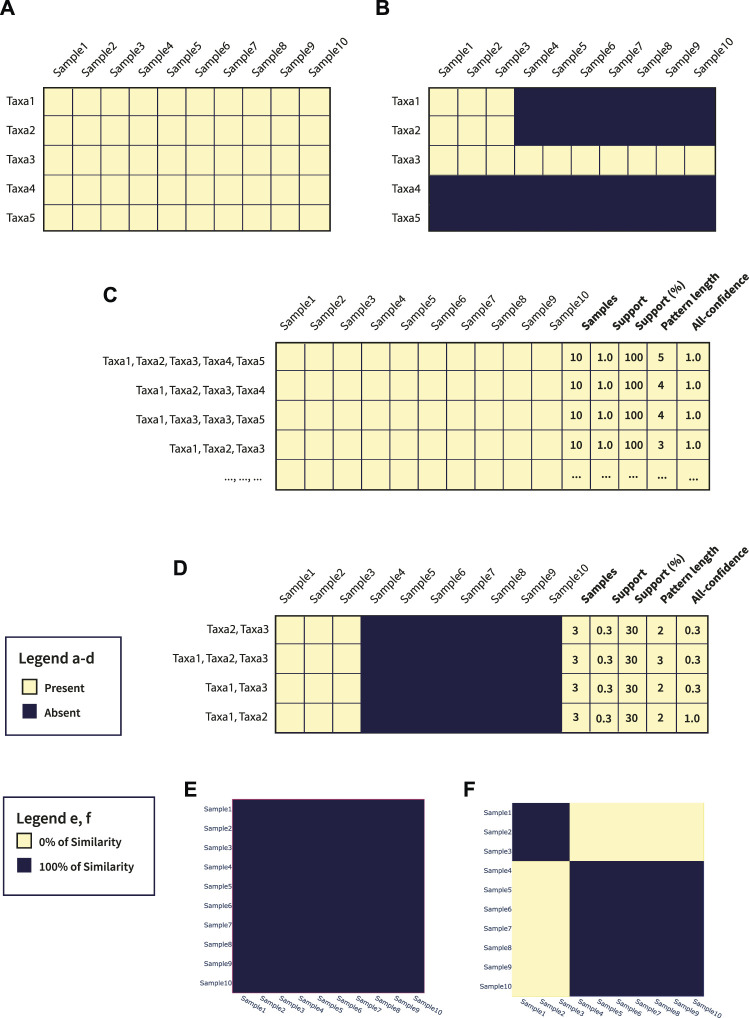
**(A)** Graphical representation of Table 1; **(B)** Graphical representation of Table 2; **(C)** Pattern table generated from Table 1; **(D)** Pattern table generated from Table 2; **(E)** Jaccard heatmap plot of Table 1; **(F)** Jaccard heatmap plot of Table 2.

To better show the potentials of microFIM, we included a demonstrative analysis of both simulated data and data belonging to real case studies (see the next Section 3). In particular, as also described in the Section 2, simulated data are composed of two main matrices with a dimension of 10 samples and 5 taxa. In Figures 3A,B a graphical representation of the simulated matrices is shown. Through microFIM, ARM analysis was performed. The final output of the analysis is the pattern table, represented in Figures 3C,D and available in Supplementary Tables S6, S7, respectively. The pattern table integrates the interest measures of length, support and all-confidence and, as it is a dataframe, patterns can be filtered and further visualized with Python libraries or other data analysis tools easily. In addition, results of the pattern table can be visualized with microFIM through the following plots: scatter plot, bar chart and heatmap. In Figures 3E,F, heatmaps built on Jaccard distance results are shown.

In detail, Dataset 1 (Figure 3A; Supplementary Table S1) is a full-presence dataset. This means that ARM can potentially generate all the combinations of patterns from a length of 1 to a length of 5. All patterns will have a 1.0 of support and a 1.0 of all-confidence, as they are all associated with each other. In this case, considering only the pattern composed by Taxa1, Taxa2, Taxa3, Taxa4, and Taxa5, with a length equal to 5 and a support equal to 1.0, can be sufficient to resume the information within the dataset. In addition, these settings can be adjusted directly by running the algorithm, avoiding the creation of uninformative patterns and reducing calculation time. In Figure 3E, Jaccard heatmap shows also the 100% similarity between Dataset 1 samples. The complete pattern list obtained by Dataset 1 is available in Supplementary Table S6.

Considering Dataset 2 (Figure 3B; Supplementary Table S2), instead, a different composition can be observed. In particular, Taxa1, Taxa2 and Taxa3 co-occur in samples 1, 2, and 3. In addition, Taxa3 is present in all the samples (Figure 3B). As we ran an ARM analysis considering a minimum length of 2, the pattern composed by only Taxa3 was not detected. However, the pattern built by Taxa1, Taxa2 and Taxa3 was detected, with a pattern length of 3 and a support of 0.3. Focus the attention on Taxa1-Taxa2 pattern, the value of all-confidence is equal to 1.0, meaning that there is a strong association between them and the rules generated from this pattern will have a minimum confidence of 1.0. Details about patterns extracted from Dataset 2 are available in Supplementary Table S7.

### 3.2 microFIM Applied on Real Case Studies

Association rule mining is a data mining technique widely used in very different research fields and applications. This chapter is dedicated to the use of ARM, in particular the pattern mining step, on real microbiome case studies. In detail, three case studies was chosen to demonstrate the potentials of ARM and microFIM: the ECAM dataset (Bokulich et al., 2016), the vaginal microbiome case study of Ravel et al. (2011) and the Montassier case study (Montassier et al., 2016) (see Section 2 for details). Considering the potential of ARM to reconstruct patterns, we focused the analysis on three main aspects: the type of input used, the filter of patterns whose elements are highly related to each other (also called hyperclique patterns; Xiong et al., 2006) and the use of metadata to filter and apply ARM.

To evaluate how ARM can be used on microbiome data, different types of inputs were considered. In particular, for the ECAM case study, we used: 1) the ECAM taxa table obtained directly from QIIME2 datasets (Bolyen et al., 2018) in which only taxa assigned to genus level, with a relative abundance > 0.1% in more than 15% of samples, are considered (Input 1—data are available in Supplementary File S3); 2) family table obtained from collapsing the original one *via* QIIME2 plugins (Input 2—Supplementary File S4); 3) a taxa table consisting only of taxa with complete taxonomy at the genus level (Input 3—Supplementary File S5).

**FIGURE 4:**
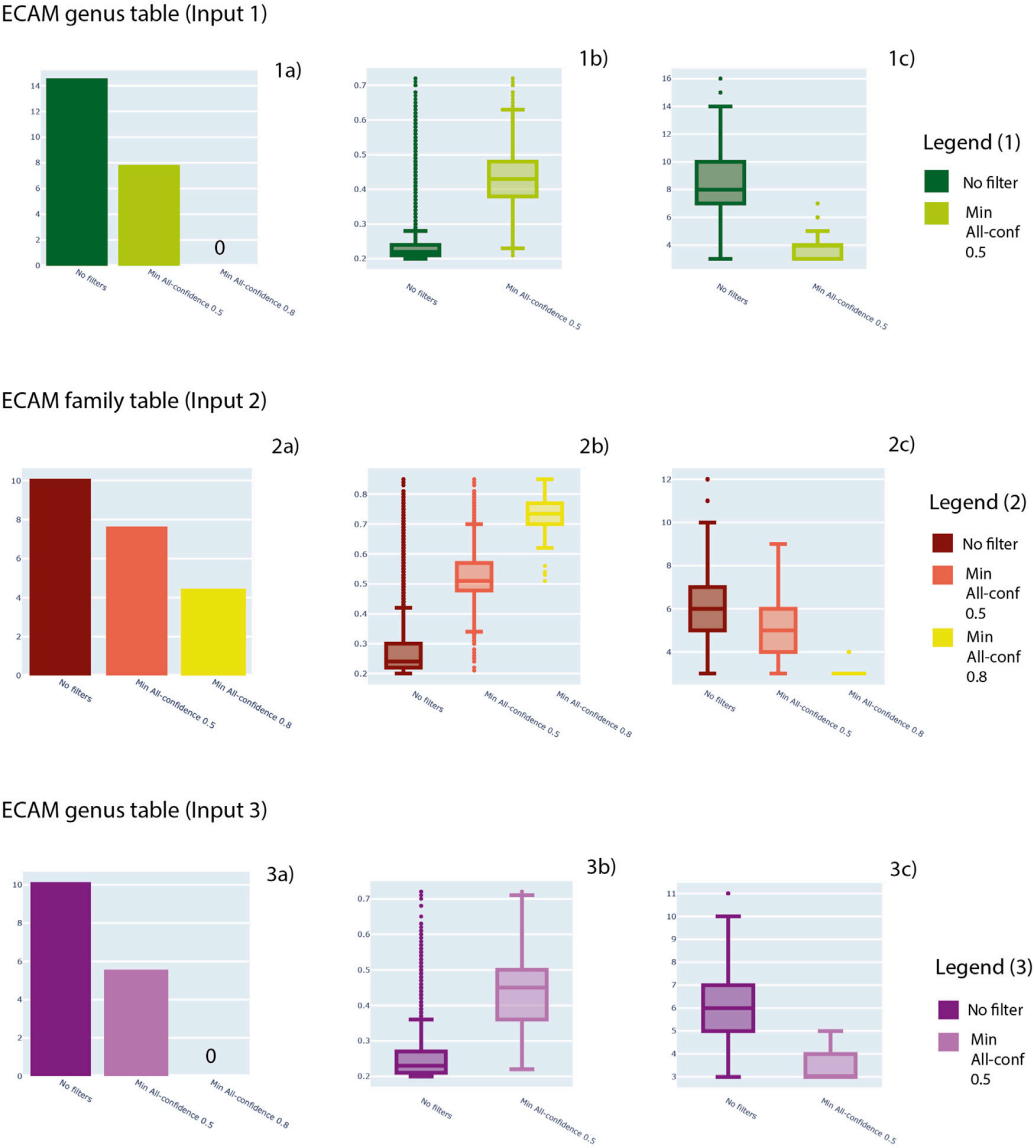
For Input 1, 2 and 3, here number of patterns obtained (1a, 2a, 3a), distribution of support values (1b, 2b, 3b) and distribution of pattern lengths (1c, 2c, 3c) are shown. In particular, three levels of analysis are shown: no filters applied to patterns, a minimum all-confidence of 0.5 and a minimum all-confidence of 0.8.

Minimum support thresholds of 0.2, minimum length of 3 and maximum length of 15 were considered. In Figure 4 we show the results about the number of patterns retrieved considering three levels of analysis: output after the analysis previously described, patterns filtered with a minimum all-confidence of 0.5 and patterns filtered with a minimum all-confidence of 0.8. In Figure 4, for each filter, the distribution of support values and pattern length are provided.

In detail, Input 1 (Supplementary File S3) generated a total of 1,844,696 patterns. The mean support achieved by the patterns generated is 0.3 and a median of 0.2, with a minimum value of 0.2 and maximum value of 0.7. Regarding the pattern length, the mean value is 8.45, while the median is 8, with a minimum value of 3 and maximum value of 16.

Family table (Input 2—Supplementary File S5) generated a total of 23,997 patterns. The mean support achieved by the patterns generated is 0.28 and a median of 0.24, with a minimum value of 0.2 and maximum value of 0.85. Regarding the pattern length, the mean value is 6.38, while the median is 6, with a minimum value of 3 and maximum value of 12.

Regarding genus table (Input 3—Supplementary File S6), ARM analysis generated a total of 25,250 patterns. The mean support achieved by the patterns generated is 0.25 and a median of 0.23, with a minimum value of 0.2 and maximum value of 0.85. Regarding the pattern length, the mean value is 6.14, while the median is 6, with a minimum value of 3 and maximum value of 11. All the results are available in Supplementary Tables S6–S8, respectively, and can be visualized in Figure 4.

In order to consider the putative informative patterns, a framework involving hypercliques patterns (Xiong et al., 2006) was applied. In particular, the all-confidence metric was considered at 0.5 and 0.8 thresholds for all the datasets analysed (Inputs 1–3).

Regarding the Input 1 (Supplementary File S3), a total of 2,213 patterns were extracted considering an all-confidence of 0.5, while no patterns were obtained with 0.8 threshold. First all-confidence threshold resulted in patterns with a mean and a median support value was 0.43, with a minimum value of 0.21 and a maximum of 0.72. Pattern length consisted in a mean of 3.9, a median length of 4, with minimum and maximum of 3 and 7, respectively.

Regarding the Input 2 (Supplementary File S4), a total of 2,081 patterns were extracted considering an all-confidence of 0.5. A mean support of 0.53 and a median support was 0.51 were observed, with a minimum value of 0.21 and a maximum of 0.85. Pattern length consisted of a mean of 4.98, a median length of 5, with minimum and maximum of 3 and 9, respectively. A total of 78 patterns were extracted considering an all-confidence of 0.8. A mean support of 0.72 and a median support was 0.73 were observed, with a minimum value of 0.51 and a maximum of 0.85. Pattern length consisted of a mean of 3.23, a median length of 3, with minimum and maximum of 3 and 4, respectively.

Regarding the Input 3 (Supplementary File S5), instead, a total of 25,250 patterns were extracted considering an all-confidence of 0.5, while no patterns were obtained with 0.8 threshold. First all-confidence threshold resulted in patterns with a mean of 0.25 and a median support value of 0.23, with a minimum value of 0.2 and a maximum of 0.72. Pattern length consisted in a mean of 6.14, a median length of 6, with minimum and maximum of 3 and 11, respectively.

For demonstrative purposes, a Jaccard heatmap considering samples belonging to the first sampling date of the ECAM dataset of the Input 3 table (Supplementary Table S5) was generated, in order to show a potential use of Jaccard distance on pattern analysis (available in Supplementary Figure S11). In general, results are summarized in Figure 4 and tables are available in Supplementary Tables S8–S10, respectively.

Overall, Input 1 obtained the highest number of patterns, achieving 1,844,696 patterns. The support distribution has a great range of values for all the three datasets, from 0.2 to almost 0.8. Also length achieved a wide range of values, considering patterns from 3 elements length to almost 16. In general, a great reduction in the number of patterns was observed considering the all-confidence filtering (Figure 4—sections 1a, 2a and 3a). In parallel, this filter resulted in higher support values (Figure 4—sections 1b, 2b and 3b) and lower pattern length (Figure 4—sections 1c, 2c and 3c).

Metadata filtering was applied to the genus ECAM dataset, considering two category types: antibiotic administration and type of delivery. The complete results of the pattern analysis are available in Supplementary Table S12. Overall, a total of 141,480 patterns were obtained from the data belonging antibiotic administration, while the opposite obtained a total of 8,223. Vaginal delivery resulted in a total of 45,412 patterns, while cesarean delivery samples resulted in 10,288. Also in this case, the usage of all-confidence filtering drastically reduced the number of explorable patterns, achieving the following results: 2 and 1 patterns for antibiotic administration and vaginal delivery, respectively, and 0 patterns for the opposites.

microFIM was also applied to other two real case studies: vaginal microbiome obtained by the work of Ravel et al. (2011) and the dataset of Montassier case study (Montassier et al., 2016). Considering the first one, different input types and metadata filtering were used: in particular, the dataset was obtained from the MLRepo collection (Vangay et al., 2019). Then, family level and genus level dataset were obtained. Dataset can be identified as Input 4 (dataset available in MLRepo; Vangay et al., 2019—Supplementary File S15A), Input 5 (dataset at the family level—Supplementary File S15B) and Input 6 (dataset at the genus level—Supplementary File S15C). As for the ECAM analysis, results are presented considering the three main input types and the number of distribution of patterns are evaluated as the previous scheme.

In particular, Input 4 (Supplementary File S15A) generated a total of 83 patterns. The mean support achieved by the patterns generated is 0.2 and a median of 0.2, with a minimum value of 0.2 and maximum value of 0.5. Regarding the pattern length, the mean value is 3.1, while the median is 3, with a minimum value of 3 and maximum value of 4. Family table (Input 5—Supplementary File S15B) generated a total of 226 patterns. The mean support achieved by the patterns generated is 0.25 and a median of 0.23, with a minimum value of 0.2 and maximum value of 0.55. Regarding the pattern length, the mean value is 3.68, while the median is 4, with a minimum value of 3 and maximum value of 6. Regarding genus table (Input 6—Supplementary File S15C), ARM analysis generated a total of 225 patterns. The mean support achieved by the patterns generated is 0.25 and a median of 0.24, with a minimum value of 0.2 and maximum value of 0.46. Regarding the pattern length, the mean value is 3.77, while the median is 4, with a minimum value of 3 and maximum value of 6. All the results are available in Supplementary Tables S15D–F, respectively, and can be consulted in Supplementary Table S14.

Minimum all-confidence of 0.5 and 0.8 were considered to evaluate hypercliques patterns. Regarding the Input 4 (Supplementary File S15A), 16 patterns were extracted considering an all-confidence of 0.5, while no patterns were obtained with 0.8 threshold. First all-confidence threshold resulted in patterns with a mean of 0.23 and a median support value was 0.21, with a minimum value of 0.2 and a maximum of 0.48. Pattern length consisted in a mean of 3.06, a median length of 3, with minimum and maximum of 3 and 4, respectively.

Input 5 (Supplementary File S15B) obtained two patterns, considering an all-confidence of 0.5, while no patterns were obtained with 0.8 threshold. The 0.5 all-confidence threshold resulted in patterns with 0.46 and 0.55 support values. Both patterns have a length of 3.

Regarding the Input 6 (Supplementary File S15C), 15 patterns were extracted considering an all-confidence of 0.5, while no patterns were obtained with 0.8 threshold. First all-confidence threshold resulted in patterns with a mean and a median support value was 0.3, with a minimum value of 0.25 and a maximum of 0.38. Pattern length consisted in a mean of 3.13, a median length of 3, with minimum and maximum of 3 and 4, respectively.

Overall, the support distribution has a low range of values for all the three input files, from 0.2 to almost 0.5. Length is around 3 elements per pattern. In general, also in this case a great reduction in the number of patterns was observed considering the all-confidence filtering (Supplementary Table S14).

Metadata filtering was applied to the dataset, considering the nugent category, low and high levels. The complete results of the pattern analysis are available in Supplementary Table S14. Overall, a total of 15,836 patterns were obtained from the data belonging to high nugent score value, while the opposite obtained a total of 21. The usage of all-confidence filtering drastically reduced the number of explorable patterns, obtaining 16 patterns for high nugent score value.

Finally, Montassier dataset (Montassier et al., 2016) was tested considering the OTU table and taxa table obtained from MLRepo pipeline (Vangay et al., 2019). A minimum support threshold of 0.9 was considered, with a minimum length of 5 and a maximum length of 10. A total of 446 patterns were obtained considering the taxa table, while 9 patterns were obtained considering the OTU table.

Distributions of pattern and length are similar between the two input files. In particular, a mean support of 0.93 and a mean length of 5.1 (5–6) were detected.

## 4 Discussion

Pattern mining strategies are now newfangled solutions for disclosure of microbial patterns (Tandon et al., 2016; Liu et al., 2021). However, besides the power of these techniques, great efforts must be undertaken to extrapolate relevant patterns that can be integrated into biological contexts (Naulaerts et al., 2015; Faust, 2021).

Basically, the strategy consists of two main phases: 1) extraction of patterns (also known as “frequent itemset mining”) and 2) rules calculation. In this work, we focused in particular on the first phase, as great potential can be achieved considering the exploration of patterns at any length and subsequently be filtered to create reliable associations.

**FIGURE 5:**
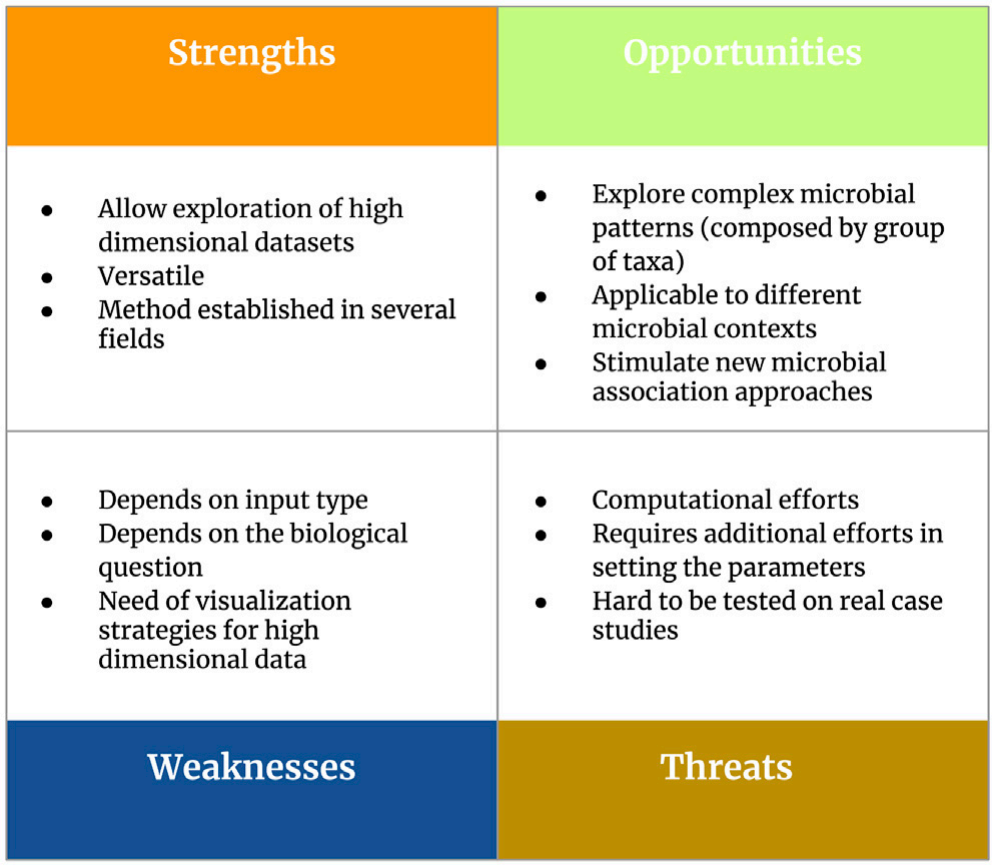
Overview of the main strengths, weaknesses, opportunities and threats (SWOT analysis) related to the use of frequent itemset mining as a tool for microbiome pattern analysis.

In detail, our Section 4 will touch two main topics: 1) considerations about parameter settings to perform pattern mining strategies in the context of 16S rRNA metabarcoding data and 2) guidelines and future perspectives to support real applications. In order to present an overview of frequent itemset mining as a tool for microbiome pattern analysis, we developed a SWOT (Strengths, Weaknesses, Opportunities, Threats) analysis (Figure 5).

### 4.1 Run Association Rule Mining Could Not Be Enough Without Care in Setting Parameters

As described above, pattern mining strategies can be powerful to get insights from large and complex datasets (Naulaerts et al., 2015). However, pattern analysis may have limitations (Faust, 2021). In this work, we provide ARM analysis on both simulated and real datasets and propose microFIM (https://github.com/qLSLab/microFIM), a Python tool specifically suited for microbiome pattern analysis. Our results will consider the pattern composition obtained through our framework (Section 2) without considering their biological implications, as it is beyond the scope of this work.

Considering the application of ARM on simulated datasets, we showed that initial settings can reduce the amount of information retrievable, both considering interest measures as support or length and all-confidence metric.

Regarding the application on the real case studies, a few considerations can be made. First of all, the type of input can change the reliability of results: different numbers of patterns have been generated considering different input types. In particular, both considering aspects related to data visualization and interpretation, the taxonomy level of investigation must be considered.

A second point that arises is the minimum support threshold to choose. The choice can be both related to biological questions, as for example which is the minimum number of samples to retain a pattern interesting, but also on technicalities. In detail, exploring all the potential patterns cannot be reliable and useful, as the number of patterns can be very high, related also to great computational efforts and visualization issues (Naulaerts et al., 2015). For this reason, we started using a support of 0.2, that means that only the taxa that co-occur in at least the 20% of samples were considered (up to 175 of 875 for the ECAM dataset and up to 77 of 388 for the Ravel case study). However, this is a case-specific threshold as no guidelines exist to set a correct support threshold in this research field. The wrong value can potentially hide information and, at the same time, create spurious patterns. In addition, it can generate misleading results without taking into account the Simpson’s paradox (Tan et al., 2002), a phenomenon in which a pattern appears frequently but disappears or drastically changes when the data are combined differently, as for example considering only a set of samples (Tan et al., 2002).

Nevertheless, once patterns are generated, filtering steps can be added, in order to both reduce the information and better evaluate specific patterns, with peculiar characteristics. Filters can include the length of patterns or additional interest measures (Agrawal et al., 1993; Karpinets et al., 2012; Naulaerts et al., 2015).

Pattern length, in particular, can be also included before running the analysis, as algorithms take into account a minimum and a maximum value of pattern length, in order to reduce the number of explorable patterns (Agrawal et al., 1993). However, this choice must be done before exploring the results. Of course, it is possible to reduce the number of patterns after extraction, but computational efforts and running time must be considered (Agrawal et al., 1993; Naulaerts et al., 2015). Pattern length can also vary based on the research field of application and the biological questions. In the ECAM case study, for example, we observed different median values of pattern length, from minimum values of 3 to maximum of 16, suggesting also different levels of analysis.

However, other metrics can be included to filter patterns (Tan et al., 2002; Omiecinski, 2003; Franceschini et al., 2012; Tang et al., 2012). Usually they are called “interest measures” and are generally used to evaluate a set of peculiar patterns, in order to filter the interesting ones (Tatti and Mampaey, 2010; Hussein et al., 2015; Naulaerts et al., 2015). Also in this case, the biological question can guide how to properly set the filtering step. In this work, we used all-confidence metrics, which generate hyperclique patterns (Omiecinski, 2003; Xiong et al., 2006). The application of this metric helps to find groups of items (in this case species or taxa) where items belonging to the same pattern are highly affiliated with each other and can generate rules with the minimum threshold chosen. Using this approach reduces drastically the number of patterns and, in addition, allows to filter only strong associated groups. In this case, the amount of information was drastically reduced considering the two thresholds of all-confidence considered (0.5 and 0.8). This reduction can promote a manual exploration of results and pave the way for exploring strong associations and putative rules. Clearly, other interest measures can be applied. All-confidence may not be the only interest measures useful for microbiome analysis. Other metrics can be selected to filter patterns, but they must be identified based on specific questions related to the research field of application (Naulaerts et al., 2015).

### 4.2 Fitting Association Rule Mining for Microbiome Studies: Guidelines to Support Real Applications

Frequent itemset mining and, subsequently, association rule mining, is a pattern mining technique able to explore items that co-occur with a certain frequency, as sets of commercial products that customers buy together in the classic supermarket basket problem (Agrawal et al., 1993; Naulaerts et al., 2015). The flexibility of frequent itemset mining techniques is demonstrated by the wide range of bioinformatics applications, from for example SNPs association studies to annotations and motif association exploration (Carmona-Saez et al., 2006; Koyuturk et al., 2006; Alves et al., 2010; Karpinets et al., 2012; Manda et al., 2012; Manda et al., 2013; Zhou et al., 2013; Agapito et al., 2015; Boutorh and Guessoum, 2016; Naulaerts et al., 2016; Manda, 2020; Ong et al., 2020). It is a powerful instrument to explore patterns from large and complex data sets (Agrawal et al., 1993; Karpinets et al., 2012; Naulaerts et al., 2015), providing different algorithms and a wide range of parameters to filter patterns of interest. Besides the most used, as support (frequency of a pattern or a rule in the dataset) or length (the number of species contained in a pattern), other metrics can be included in the pattern analysis (Naulaerts et al., 2015; Agrawal et al., 1993; Hornik et al., 2005). Beside its potentials, great efforts have to be made to perform pattern mining strategies on microbiome data and obtain reliable and interpretable results, with sound biological implications. As mentioned above, a few points raised from the works done. From threshold choices to input data types, setting pattern analysis is not an easy task. Considering the peculiarities of microbiome data and the flexibility of the technique, here we propose five statements to guide researchers before starting ARM analysis.

#### 4.2.1 Setting the Input Data

This point highlights the importance of the type of pattern to be considered. In the microbial ecology field, a lot of interest probably regards the investigation of species patterns, in order to evaluate community patterns and putative ecological processes. However, this is not straightforward if we consider 16S rRNA metabarcoding data: taxonomy does not always reach a species level and this uncertainty can negatively impact pattern reconstruction. In addition, noise derived from contamination or sequencing biases can be present (Faust and Raes, 2012; Balint et al., 2016; Gloor et al., 2017; Faust, 2021). However, precautions can be taken: removing uncertain taxa or cleaning the table based on abundance thresholds or statistical methods is possible (Faust and Raes, 2012; Balint et al., 2016; Gloor et al., 2017). Different levels of taxonomy can be used as input, as we also demonstrated in the previous sections. Of course, choices must be taken with conscience as they will impact on the final result and therefore the interpretation must be correctly contextualized.

#### 4.2.2 Consider the Use of Metadata

The inclusion or filtering considering metadata information can improve the reliability of the method, both looking for specific patterns linked to metadata and also to better explore the dataset. In this way, we can reduce the information to be explored, lowering the support value, retaining rare or patterns related to specific metadata, and preventing Simpson’s paradox issues (Agrawal et al., 1993; Naulaerts et al., 2015).

#### 4.2.3 Individuate What is Interesting for the Specific Case Study

The definition of what is interesting depends on the biological context at issue. No simple guidelines exist, as the application of pattern mining on microbiome data is still in its infancy (Naulaerts et al., 2015). Testing and developing new metrics is an important field of research and can make a difference to track reliable patterns that can be further used for classification tasks or functional analysis. In this work, we applied the all-confidence metric (Omiecinski, 2003; Xiong et al., 2006). However, we believe that other interest measures can be applied and a wide variety of them are available in other tools already developed (Hahsler et al., 2005; Hahsler et al., 2011). In general, this step allows to drastically reduce the number of explorable patterns (Tan et al., 2002; Omiecinski, 2003; Xiong et al., 2006).

Basically, length can be used to clean the information extracted via ARM. As ARM can generate patterns at any length, single items or only pairs of items can be pruned, in order to find interesting associations composed by 3 or more elements. From a biological point of view, exploring longer microbial patterns can enhance microbial community investigations and pave the way for high-order interactions exploration (Faust, 2021).

#### 4.2.4 Consider Computational Time

As fully described in previous works, data dimensions and density drastically increase time calculation and memory usage (Agrawal et al., 1993; Naulaerts et al., 2015). Reducing input data can make ARM more reliable and faster to be performed (Agrawal et al., 1993; Naulaerts et al., 2015). In addition, beside the common concept of pattern, closed and maximal patterns exist. Both result in a faster extraction, but with a reduction of information (Agrawal et al., 1993; Naulaerts et al., 2015).

Overall, the inclusion of interest measures directly into the ARM framework may favour the development of new faster algorithms, leading the technique directly to the exploration of specific patterns (Omiecinski, 2003; Xiong et al., 2006; Naulaerts et al., 2015).

#### 4.2.5 Tools and Visualization Strategies

To better suit pattern mining for microbiome data applications, tools and visualization techniques are essentials (Naulaerts et al., 2015). In detail, in this work we tried to concept a new pattern mining output combining the common microbiome output with pattern analysis. The pattern table can be an important resource to perform and visualize pattern results in a microbial perspective. In addition, it allows further statistical analysis that is usually performed for microbiome data. Considering the visualization process, we set up different plots to have an overview of pattern distributions and create a Jaccard matrix to show the distance between samples. However, different visualization methods exist, based on tables, matrices and graphs (Naulaerts et al., 2015). Here we cite the R packages arulesviz, FPViz and WiFIsViz (Hornik et al., 2005; Hahsler et al., 2011; Naulaerts et al., 2015). Even though these visualizations allow different strategies to explore data, issues related to high dimensional dataset remain and none of them are conceptualized for microbiome analysis. At the same time, collecting human readable information can facilitate data visualization strategies and interpretation (Naulaerts et al., 2015), but of course interesting measures must be considered. Finally, considering practicality of use, several ARM implementations can be utilized (Naulaerts et al., 2015). Moreover, frameworks have been implemented, often accompanied by GUI (Graphical User Interface) or interactivity components (Naulaerts et al., 2015). However, a deepening in the microbiome field has not been established yet.

#### 4.2.6 Evaluation and Benchmarking Strategies

From a computational point of view, the complexity and dynamics of microbial communities leads to difficulties in developing and testing methods to evaluate them. In general, it was demonstrated that microbial co-occurrence analysis may be an extraordinarily promising approach for studying microbiomes (Faust and Raes, 2012). Several works explained how co-occurrences reveal indications about ecological processes shaping community structure (Lima-Mendez et al., 2015), exploring hub species and potential microorganisms relationships (Berry and Widder, 2014). Further, Ma et al. (2020) showed how global microbial co-occurrence analysis and network reconstruction may be an encouraging strategy to reveal patterns and explore new mechanisms. However, besides these results, transform microbiome data into purposeful biological insights remain challenging, as also demonstrated by different evaluations (Faust and Raes, 2012; Berry and Widder, 2014), and open questions still remain (Faust and Raes, 2012; Layeghifard et al., 2017; Ma et al., 2020; Faust, 2021). The use of ARM on microbiome data models or datasets created in-silico will be necessary to disentangle the potentials of ARM in the microbiome research field, also considering the range of microbiome aspects that can be considered (Weiss et al., 2016; Hosoda et al., 2020; Faust, 2021). In particular, tests should examine how the technique is affected by noise signals, both related to sequencing and laboratory protocols (Weiss et al., 2016). In addition, as microbiome data may potentially describe a complex and intricate ecological community, several ecological aspects can be evaluated with ARM, both describing the generation of redundant information and the difficulty associated with extracting patterns due to specific ecological behaviors, as for example competition, exclusion or symbiosis (Faust and Raes, 2012; Weiss et al., 2016; Faust, 2021).

In general, recent advancements in data integration and data reuse strategies may enhance the exploration of microbial patterns from large-scale studies (Jordan and Mitchell, 2015; Ma et al., 2020; Su et al., 2020; Ghannam and Techtmann, 2021). Microbiome simulators and *in vitro* studies can be a great instrument for benchmarking works and improve guidelines to apply ARM (Faust, 2021). Beside the potential of ARM on large scale analysis, giving a great overview of data under investigation (Naulaerts et al., 2015), these advancements may contribute to developing tests and benchmarking strategies in order to set ARM for microbial pattern research looking at biological implication, specifically.

Concluding, all the challenges mentioned above can disentangle ARM analysis for microbiome pattern exploration. As the output of the analysis can be extensive and redundant, results should be interpreted with caution. The associations extracted do not necessarily imply causality. Instead, it suggests a strong co-occurrence relationship between species. Causality, on the other hand, requires knowledge about the causal and effect attributes in the data (Tan et al., 2002). There are several approaches to evaluate the robustness of an output. In this first work, pattern length, support and all-confidence were explored and included in the microFIM tool. From a biological perspective, filtering results with these parameters could help to highlight meaningful patterns, but may not be enough. Further, we tried to depict issues that we think must be considered before using an ARM approach for specifical biological traits. As there is an interest in research to exploit data mining techniques, citing for example the works of Srivastava et al., 2019 or Zakrzewski et al., 2017, we also think that suiting ARM for microbiome analysis will be a great resource in the future. Considering the huge amount of data available and produced with the advent of High-Throughput DNA Sequencing (HTS) technologies, an increasing selection of large-scale data science strategies seems to have enormous potential in resolving challenges in microbiome pattern exploration (Jordan and Mitchell, 2015; Kypides et al., 2016). Association rule mining and microFIM tools may have great potential not only with 16S rRNA metabarcoding data, but also in a wide range of applications. As also supported by Naulaerts et al. (2016), ARM analysis is a versatile technique: the integration of files such as taxa tables guarantees the usage also on a wide variety of datasets belonging from different sources, as for example the QIITA platform (https://qiita.ucsd.edu/; Gonzales et al., 2018) or the MLrepo (https://knights-lab.github.io/MLRepo/; Vangay et al., 2019), but not only. Beside the main focus of this work and microFIM development, very different types of data can be analysed and integrated with ARM framework. From gene associations to merely metabarcoding projects, whose output has the same structure of 16S rRNA taxa table, microFIM may potentially pave the way for multiple usages, creating a bridge with several research fields and applications.

## Data Availability

The original contributions presented in the study are included in the article/Supplementary Material, further inquiries can be directed to the corresponding author.
