# Supplementary material for: Extending Association Rule Mining to Microbiome Pattern Analysis: Tools and Guidelines to Support Real Applications
Source: Front Bioinform. 2022 Jan 10;1:794547. doi: 10.3389/fbinf.2021.794547 (PMC9580939; doi:10.3389/fbinf.2021.794547)
Supplement: Supplementary file 1 [file DataSheet1.pdf]

Supplementary Material

1. Supplementary Table 1

Table describing a simulated dataset 1 composed of 5 taxa and 10 samples (CSV format).

2. Supplementary Table 2

Table describing a simulated dataset 2 composed of 5 taxa and 10 samples (CSV format).

3. Supplementary Table 3

ECAM taxa table obtained directly from QIIME2 datasets (Bolyen et al., 2019) in which only taxa assigned to genus level, with a relative abundance > 0.1 % in more than 15% of samples, are considered (TSV format).

4. Supplementary Table 4

Family ECAM taxa table obtained collapsing the ECAM dataset (Supplementary Table 3; Bolyen et al., 2019) to the family level via QIIME2 plugins (https://github.com/qiime2/q2-taxa) (TSV format).

5. Supplementary Table 5

Genus ECAM taxa table obtained collapsing the ECAM dataset (Supplementary Table 3; Bolyen et al., 2019) consisting only of taxa with complete taxonomy at the genus level (TSV format).

6. Supplementary Table 6

Pattern table generated performing microFIM on simulated dataset 1 (Supplementary Table 1) with the minimum support of 0.3, a minimum length of 2 and a maximum length of 10 (CSV format).

7. Supplementary Table 7

Pattern table generated performing microFIM on simulated dataset 2 (Supplementary Table 2) with the minimum support of 0.3, a minimum length of 2 and a maximum length of 10 (CSV format).

8. Supplementary Table 8

Table generated performing microFIM on ECAM dataset (Supplementary Table 3) with a minimum support of 0.2, a minimum length of 3 and a maximum length of 15 (CSV format).

9. Supplementary Table 9

Pattern table generated performing microFIM on ECAM dataset at family level (Supplementary Table 4) with a minimum support of 0.2, a minimum length of 3 and a maximum length of 15 (CSV format).

10. Supplementary Table 10

Pattern table generated performing microFIM on ECAM dataset at genus level (Supplementary Table 5) with a minimum support of 0.2, a minimum length of 3 and a maximum length of 15 (CSV format).

11. Supplementary Figure 11

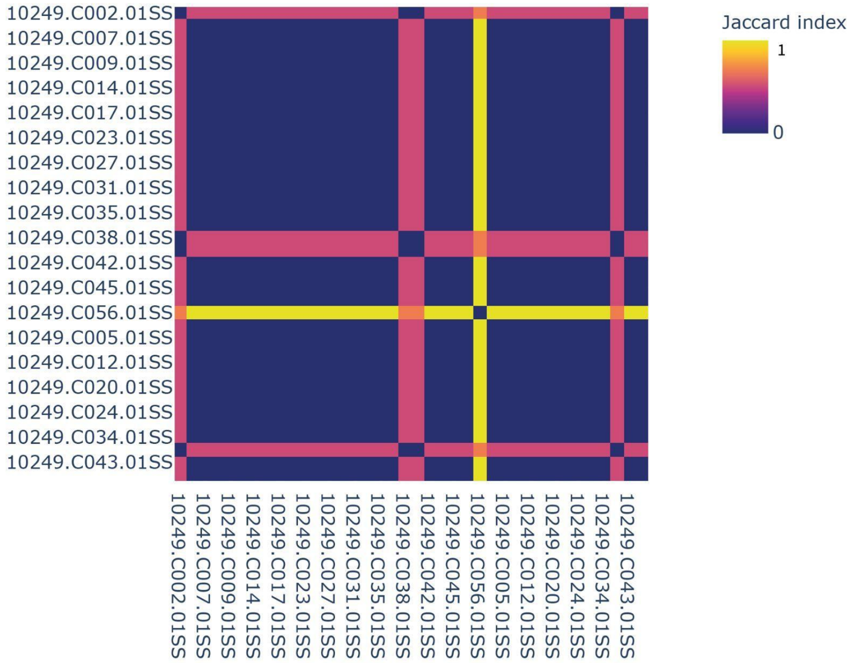

Supplementary Figure 11. Heatmap representing Jaccard distance matrix was generated via microFIM visualization phase on the ECAM dataset, considering Input 3 and samples belonging to the first sampling date.

12.

13.

14. Supplementary Table 12

The table below describes the results regarding the analysis of patterns extracted from the ECAM dataset (Bokulich et al., 2016). In particular, the table obtained with QIIME2 pipeline at the genus

level was considered (details in Material and methods section). Metadata considered to filter the samples were antibiotic administration (yes or no) and type of delivery (cesarean and vaginal). In the table, support and pattern length distributions were reported as mean and minimum and maximum values. In Input columns, parenthesis represent the dataset available in the Supplementary File 15. Complete descriptions are provided in the next section.

Input	N. of patterns	Support	Length
Antibiotic yes (13a)	141,480	0.26 (0.2-0.77)	7.52 (3-14)
Antibiotic yes - allc 0.8	2	0.58, 0.58	3
Antibiotic no (13b)	8,223	0.25 (0.2-0.9)	5.35 (3-10)
Antibiotic no - allc 0.8	0	/	/
Vaginal delivery (13c)	45,412	0.25 (0.2-0.76)	6.57 (3-12)
Vaginal delivery - allc 0.8	1	0.76	3
Cesarean delivery (13d)	10,288	0.24 (0.2-0.72)	5.41 (3-10)
Cesarean delivery - allc 0.8	0	/	/

15. Supplementary File 13

ECAM datasets obtained by filtering with metadata (antibiotic usage and type of delivery) are available in Supplementary File 13 (zip). Below a description of all the files included.

1. Supplementary File 13a

Table generated performing microFIM on genus ECAM dataset considering samples with antibiotic administration. A minimum support of 0.2, a minimum length of 3 and a maximum length of 15 were used (CSV format).

2. Supplementary File 13b

Table generated performing microFIM on genus ECAM dataset considering samples with no antibiotic administration. A minimum support of 0.2, a minimum length of 3 and a maximum length of 15 were used (CSV format).

3. Supplementary File 13c

Table generated performing microFIM on genus ECAM dataset considering samples with vaginal delivery metadata. A minimum support of 0.2, a minimum length of 3 and a maximum length of 15 were used (CSV format).

4. Supplementary File 13d

Table generated performing microFIM on genus ECAM dataset considering samples with cesarean delivery metadata. A minimum support of 0.2, a minimum length of 3 and a maximum length of 15 were used (CSV format).

5. Supplementary File 13e

Metadata file of ECAM dataset (TSV format).

16. Supplementary Table 14

The table below describes the results regarding the analysis of patterns extracted from the Ravel dataset (Ravel et al., 2011). In particular, the table obtained with the MLRepo pipeline and family and genus level was considered. Metadata considered to filter the samples at the genus level were Nugent Score equal to low and Nugent Score equal to high. In the table, support and pattern length distributions were reported as mean and minimum and maximum values. In Input columns, parenthesis represent the dataset available in the Supplementary File 15. Complete descriptions are provided in the next section.

Input	N. of patterns	Support	Length
Original (15d)	83	0.2 (0.2-0.5)	3.1 (3-4)
Original - allc > 0.5	16	0.23 (0.2-0.48)	3.06 (3-4)
Original - allc > 0.8	0	/	/
Family (15e)	226	0.25 (0.2-0.55)	3.68 (3-6)
Family - allc > 0.5	2	0.25, 0.38	3
Family - allc > 0.8	0	/	/
Genus (15f)	225	0.25 (0.2-0.46)	3.77 (3-6)
Genus - allc > 0.5	15	0.3 (0.25-0.38)	3.13 (3-4)
Genus - allc > 0.8	0	/	/
Nug. low (15g)	21	0.23 (0.2-0.29)	3.14 (3-4)
Nug. low - allc > 0.8	0	/	/

Nug. high (15h)	15,836	0.26 (0.2-0.9)	5.65 (3-11)
Nug. high - allc > 0.8	16	0.82 (0.78-0.9)	3.19 (3-4)

17. Supplementary File 15

Ravel (2011) taxa tables and datasets obtained by filtering with metadata (nugent score equal to low and high) are available in Supplementary File 15 (zip). Below a description of all the files included.

1. Supplementary File 15a

Taxa table of the Ravel dataset (Ravel et al., 2011) available in MLRepo (Vangay et al., 2019; CSV format).

2. Supplementary File 15b

Taxa table obtained collapsing the Ravel dataset (Ravel et al., 2011) at the family level (CSV format).

3. Supplementary File 15c

Taxa table obtained collapsing the Ravel dataset (Ravel et al., 2011) at the genus level (TSV format).

4. Supplementary File 15d

Table generated performing microFIM on Supplementary File 15a. A minimum support of 0.2, a minimum length of 3 and a maximum length of 15 were used (CSV format).

5. Supplementary File 15e

Table generated performing microFIM on Supplementary File 15b. A minimum support of 0.2, a minimum length of 3 and a maximum length of 15 were used (CSV format).

6. Supplementary File 15f

Table generated performing microFIM on Supplementary File 15c. A minimum support of 0.2, a minimum length of 3 and a maximum length of 15 were used (CSV format).

7. Supplementary File 15g

Table generated performing microFIM on genus Ravel dataset considering samples with nugent score equal to low. A minimum support of 0.2, a minimum length of 3 and a maximum length of 15 were used (CSV format).

8. Supplementary File 15h

Table generated performing microFIM on genus Ravel dataset considering samples with nugent score equal to high. A minimum support of 0.2, a minimum length of 3 and a maximum length of 15 were used (CSV format).

9. **Supplementary File 15i**

Metadata file of Ravel dataset (also available in MLRepo; Vafngay et al., 2019) (CSV format).

18. **Supplementary File 16**

The table below describes the results regarding the analysis of patterns extracted from the Montassier dataset (Montassier et al., 2016). In particular, the OTU table obtained with the MLRepo pipeline and taxa table were considered. In the table, support and pattern length distributions were reported as mean and minimum and maximum values. In Input columns, parenthesis represent the dataset available in the Supplementary File 17. Complete descriptions are provided in the next section.

Input	N. of patterns	Support	Length
Otu table (17a)	9	0.93	5.1 (5-6)
Taxa table (17b)	446	0.93	5.1 (5-6)

19. **Supplementary File 17**

Montassier (2016) taxa and OTU datasets are available in Supplementary File 17 (zip). Below a description of all the files included.

1. **Supplementary File 17a**

OTU table of the Montassier case study (Montassier et al., 2016) also available in MLRepo (Vangay et al., 2019; TSV format).

2. **Supplementary File 17b**

Taxa table of the Montassier case study (Montassier et al., 2016) also available in MLRepo (Vangay et al., 2019; TSV format).

3. **Supplementary File 17c**

Table generated from the Montassier OTU dataset (Montassier et al., 2016), obtained with a minimum support of 0.9, a minimum length of 5 and a maximum length of 10 (CSV format).

4. **Supplementary File 17d**

Table generated from the Montassier taxa dataset (Montassier et al., 2016), obtained with a minimum support of 0.9, a minimum length of 5 and a maximum length of 10 (CSV format)

5. **Supplementary File 17e**

Metadata of the Montassier case study (Montassier et al., 2016) also available in MLRepo (Vangay et al., 2019; TSV format).
