# Supplementary figures and images for: Extending Association Rule Mining to Microbiome Pattern Analysis: Tools and Guidelines to Support Real Applications
Source: Front Bioinform. 2022 Jan 10;1:794547. doi: 10.3389/fbinf.2021.794547 (PMC9580939; doi:10.3389/fbinf.2021.794547)

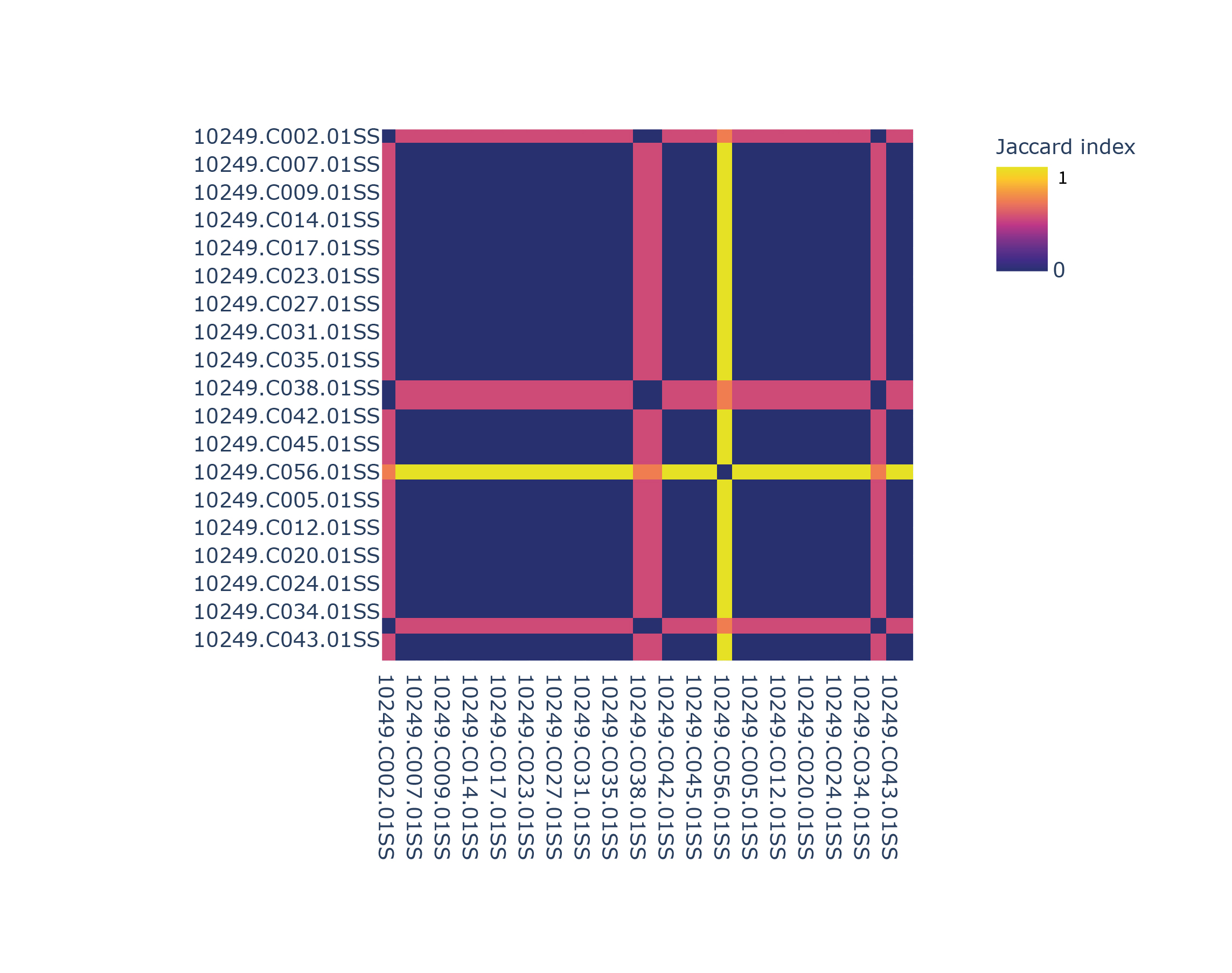

Supplement: Supplementary file 2 [file DataSheet2.zip › supplementary_data/Supplementary_figure_11.jpg]
